# Electrochemotherapy of Deep-Seated Tumors: State of Art and Perspectives as Possible “EPR Effect Enhancer” to Improve Cancer Nanomedicine Efficacy

**DOI:** 10.3390/cancers13174437

**Published:** 2021-09-03

**Authors:** Maria Cristina Bonferoni, Giovanna Rassu, Elisabetta Gavini, Milena Sorrenti, Laura Catenacci, Maria Luisa Torre, Sara Perteghella, Luca Ansaloni, Marcello Maestri, Paolo Giunchedi

**Affiliations:** 1 Department of Drug Sciences, University of Pavia, Viale Taramelli 12, 27100 Pavia, Italy; mariacristina.bonferoni@unipv.it (M.C.B.); milena.sorrenti@unipv.it (M.S.); laura.catenacci@unipv.it (L.C.); marina.torre@unipv.it (M.L.T.); sara.perteghella@unipv.it (S.P.); 2 Department of Chemistry and Pharmacy, University of Sassari, Via Muroni 23/a, 07100 Sassari, Italy; grassu@uniss.it (G.R.); eligav@uniss.it (E.G.); 3 IRCCS Policlinico San Matteo Foundation and Department of Clinical-Surgical, Diagnostic and Paediatric Sciences, University of Pavia, 27100 Pavia, Italy; l.ansaloni@smatteo.pv.it (L.A.); mmaestri@smatteo.pv.it (M.M.)

**Keywords:** electroporation, electrochemotherapy, deep-seated tumors, EPR effect, EPR enhancer, cancer nanomedicine, bleomycin, cisplatin

## Abstract

**Simple Summary:**

Electroporation-based therapies (reversible electroporation, irreversible electroporation, electrochemotherapy) are used for the selective treatment of deep-seated tumors. The combination of the structural modifications of the lipid bilayer of cell membranes, due to the application of electrical pulses in the targeted tissue, with the concomitant systemic (intravenous) administration of drugs can be considered as a sort of bridge between local-regional and systemic treatments. A possible further application of these techniques can be envisaged in their use as enhancers of the so-called “enhanced permeability and retention” effect. The intratumoral uptake of drug-loaded nanocarriers concomitant with the application of electric pulses in the target tumor is a new scenario worthy of attention and can represent a potential new frontier for drug delivery in oncology.

**Abstract:**

Surgical resection is the gold standard for the treatment of many kinds of tumor, but its success depends on the early diagnosis and the absence of metastases. However, many deep-seated tumors (liver, pancreas, for example) are often unresectable at the time of diagnosis. Chemotherapies and radiotherapies are a second line for cancer treatment. The “enhanced permeability and retention” (EPR) effect is believed to play a fundamental role in the passive uptake of drug-loaded nanocarriers, for example polymeric nanoparticles, in deep-seated tumors. However, criticisms of the EPR effect were recently raised, particularly in advanced human cancers: obstructed blood vessels and suppressed blood flow determine a heterogeneity of the EPR effect, with negative consequences on nanocarrier accumulation, retention, and intratumoral distribution. Therefore, to improve the nanomedicine uptake, there is a strong need for “EPR enhancers”. Electrochemotherapy represents an important tool for the treatment of deep-seated tumors, usually combined with the systemic (intravenous) administration of anticancer drugs, such as bleomycin or cisplatin. A possible new strategy, worthy of investigation, could be the use of this technique as an “EPR enhancer” of a target tumor, combined with the intratumoral administration of drug-loaded nanoparticles. This is a general overview of the rational basis for which EP could be envisaged as an “EPR enhancer” in nanomedicine.

## 1. Introduction

Globally, cancer is the leading cause of death in advanced countries. Its incidence increases with the aging of the population, and it is considered the biggest limit to the expectancy of life duration in the 21st century, as globally, mortality rates determined by cancer are increasing [1].

Surgical treatment with tumor removal is the best approach, but its success depends on early diagnosis and the absence of metastases. Chemotherapy and radiotherapy represent further tools available against cancer. However, conventional chemotherapy has some limits, mainly represented by the lack of tumor selectivity. Therefore, there is the need to develop new tools able to target tumors with high selectivity, to improve early diagnostics and the efficacy of anticancer drugs and reduce drug toxicity [2].

In 1986, Maeda and Matsumura discovered the enhanced permeability and retention (EPR) effect, and this was the starting point of many studies about the possible use of nanomedicines in cancer chemotherapy [3,4]. The EPR effect is a phenomenon observed in solid tumors determined by the peculiar anatomical and physio-pathological characteristics constituted by fenestrated tumor vasculature and reduced lymphatic recovery [5]. When nanomedicines are administered systemically, the hyperpermeable tumor vasculature determines their leaking from the tumor blood vessels into the tumor interstitium, a phenomenon that does not occur in healthy vessels, while poor tumor lymphatic drainage contributes to enhanced retention and accumulation of nanoparticles [6]. This phenomenon is classified as a form of passive targeting of nanoparticles into the tumor body [6,7] and it has been the starting point for the use of nanocarriers in EPR-based tumor targeting [8]. Over the years, research has been concentrated on the development of cancer nanomedicines, resulting in thousands of publications [9]. Different kinds of nanomedicines can be prepared, such as polymeric [7,10,11,12,13,14,15] and dendrimeric [16,17] nanoparticles, solid lipid nanoparticles (SLNs) [18,19,20], liposomes [20], and nanomicelles [21].

However, in the last few years, many studies have shown that in human advanced cancers, there is strong heterogeneity of the EPR effect [22]. For example, Ding et al. showed that about 90% of human renal tumors have an EPR effect that is significantly heterogeneous [23]. It has been claimed that only about 1% of the dose of nanosized formulations reaches the tumor after intravenous administration [24]. Danhier wrote an interesting review about the present situation of cancer nanomedicine and this author claims that the EPR effect works in rodents but not in humans, that tumor targeting has not been proved in the clinic, and that the rationale of anticancer nanomedicine based on EPR is failing [25]. A recent “cover story” written by Park is significantly entitled “The beginning of the end of the nanomedicine hype” [26].

The heterogeneous and unpredictable EPR effect of human tumors explains why there is a great difference between the large number of preclinical research papers dealing with anticancer nanomedicines and the relatively small number of anticancer nanomedicine products reaching clinical studies and then the market [27]. At the preclinical stage, most anticancer nanomedicine studies are limited to animal tests and only a few of them reach early phase clinical trials. A critical aspect is represented by the animal models by which nanomedicines (polymeric nanoparticles, SLNs, etc.) are preliminarily studied in vivo. Murine tumors have a different microenvironment with respect to human ones because most rodent tumors grow much faster [25,28]. The EPR effect is more predictable in small-animal models of xenograft tumors that are utilized for the evaluation of anticancer nanomedicines, with respect to human tumors [29]. Therefore, the preliminary, often encouraging, results obtained in animal models, in most cases, cannot be translated to humans.

A possible reason for the insufficient results of the EPR effect in humans is that human tumors are often diagnosed when they are larger than 3 mm and up to 10 cm or more and such large tumors can present with occluded blood vessels owing to vascular clots and thrombi and consequently partially suppressed blood flow [22]. Islam et al. claimed that this blood flow suppression is a key feature of late-stage cancers and, consequently, determines “little or no drug delivery and, therefore, a highly limited EPR effect” after systemic administration of the nanomedicine [22].

In accordance with this explanation, Fang et al. claimed that advanced-stage/large tumors are characterized by a heterogeneous structure and consequently show a heterogeneous EPR behavior, while early stage/small tumors have a more uniform EPR effect [5]. The result is an “inadequate EPR effect”, which means an irregular and unpredictable accumulation of the nanomedicines into the tumor body, which is particularly below the expectations in the case of deep-seated tumors [5].

Therefore, according to Fang’s considerations, there is a strong need for “EPR enhancers” to improve cancer nanomedicine’s efficacy [5]. Many strategies are involved to achieve this goal. Dhaliwal and Zheng defined the so-called “EPR-adaptive delivery”, which is an ensemble of techniques characterized by the capacity of modifying tumor accessibility and that are applied to improve the EPR effect [30]. Shi et al. classified the techniques that are used to modify the tumor microenvironment in so-called “pharmacological co-treatments” and “physical co-treatments” [27]. Angiotensin II (AT-II)-induced hypertension [31], NO-generating agents [32], and CO generating agents [33] are examples of the use of the pharmacological approach. According to Dhaliwal and Zheng, physical techniques involve the use of external stimuli, which can be ultrasound, radiation, hyperthermia, or photodynamic therapy, to modify the microenvironment of the tumor [30]. Selected examples of EPR enhancers are reported in Table 1.

The electroporation techniques have characteristics that make them potentially interesting for use as a promoter of the EPR effect. In the last years, some works have shown in deep-seated tumors an improvement of the uptake of nanocarriers containing antitumoral drugs, with the concomitant application of an electroporation technique.

This review is a general overview concerning the reasons for which EP could be considered as a potential “EPR enhancer” for local (intratumoral) administration of drug-loaded nanomedicines.

## 2. Electroporation-Based Therapies

When consecutive electrical pulses are applied at a cellular level, they interact with the cellular membrane, determining structural modifications. Depending on the amplitude and number of pulses applied, these modifications can be permanent, so-called “irreversible electroporation” (IRE), or reversible, so-called “electroporation” (EP) [34].

In IRE, the use of short electric pulses at high intensity (at least 10 times with respect to those utilized in the reversible EP) determines cell necrosis. IRE can be used as a nonthermal ablative technique to treat tumors that are unsuitable for surgery or thermal ablation owing to their anatomic location or even during open surgery to complete the surgical treatment [35,36]. IRE changes cell membranes only; no other structure, such as the supportive stroma, is modified. Therefore, the preservation of the components of the connective tissue enhances the structural integrity of the tissues and enhances the local ablation. This makes IRE attractive for tumors close to blood vessels, and biliary or urinary tracts [37]. Many studies have also showed the preservation of nerves, as the endo-neural architecture is left intact after IRE treatment [38,39].

Clinical applications of IRE are growing, especially for the treatment of liver and pancreatic cancers, and show that IRE represents a safe therapeutic approach, and that the application of this technique may improve patient survival and quality of life [40,41,42,43,44]. Furthermore, applications in the therapy of other tumors are reported. A recent study about the clinical impact of IRE for the treatment of unresectable hilar cholangiocarcinoma showed that IRE is capable of controlling its local progression [45]. In the treatment of prostate cancer, it is reported that IRE has a comparable efficacy to radical prostatectomy [46,47].

In EP, the application of the external electric pulses determines a reversible enhancement of the permeability of the cell membrane. The theory of “aqueous pore formation” explains the mechanisms involved. When an electric field (>50 V) is applied on the cell membranes, their surface tension is destabilized, and molecules can pass into the cytosol by the spontaneous formation of aqueous pores [34]. It is supposed that the voltage applied determines a rearrangement of the lipidic components of the bilayer, which determines the pore formation. The hypothetical formation of the pores caused by EP has not been observed yet as they are quickly reversible and small [48]. According to another theory, there are no actual holes, but the improvement of permeability is due to the reversible loss of the ordered structure of the lipid bilayer determined by the increase in the transmembrane voltage [49].

Whatever the explanation, this reversible improvement of permeability can be used for a therapeutic purpose. If local application in a target tissue is combined with systemic or the local administration of a drug with a low capacity for permeation through the cell membrane, this capacity is locally improved. Thus, the drug can come inside the cell and can explain its therapeutic effect, especially where an electric field is applied [43]. The result is drug targeting.

Suitable candidates are molecules too big to pass through the membrane of the cells, such as nucleic acids (DNA, RNA) [50]. For these reasons, EP has been used for in vivo gene delivery and has proved to be effective in transgene expression, vaccine production, and enzyme replacement [50,51].

If the poorly permeable drug used is an anticancer agent, the technique is defined as electrochemotherapy (ECT) [52].

One of the first examples of ECT involved bleomycin as the drug [53]. Here, the target tissue is a tumor, and the local application of the electric pulses increases the drug permeation into the tumoral cells, especially at the site of the electric pulse application. This technique can be also applied intraoperatively (open surgery) to treat the operative margins after the resection of the tumoral nodule because the tissues around the nodule are often infiltrated by tumoral cells, a cause of relapse [49].

The agents that can be used for ECT are non-permeant or poorly permeant chemotherapeutic drugs that, in this way, greatly increase their efficacy [48]. The antitumoral action of ECT is not determined only by the increased uptake of the antitumoral drugs. It is reported that ECT stimulates the immune response [54], as after the death of electroporated tumoral cells, cancer antigens are recognized by dendritic cells, and this can increase the antitumoral effect [55].

As for IRE, also with ECT, the structural integrity of the target tissue is maintained, even if this also depends on the dose of the drug administered. A radiological study by Brloznik et al. showed no clinically significant damage of porcine liver after ECT with bleomycin. The treated areas were characterized by intact walls of the vessels and patency, and no thrombi or hemorrhage were observed [56]. For this reason, Probst et al. reported that ECT can determine lower side effects in the patients with respect to traditional systemic chemotherapy [52].

ECT is utilized, even in a clinical routine, for the treatment of cutaneous and subcutaneous tumors [52,57] and the international guidelines for both primary and metastatic skin cancer treatment include this technique [58].

**Figure 1:**
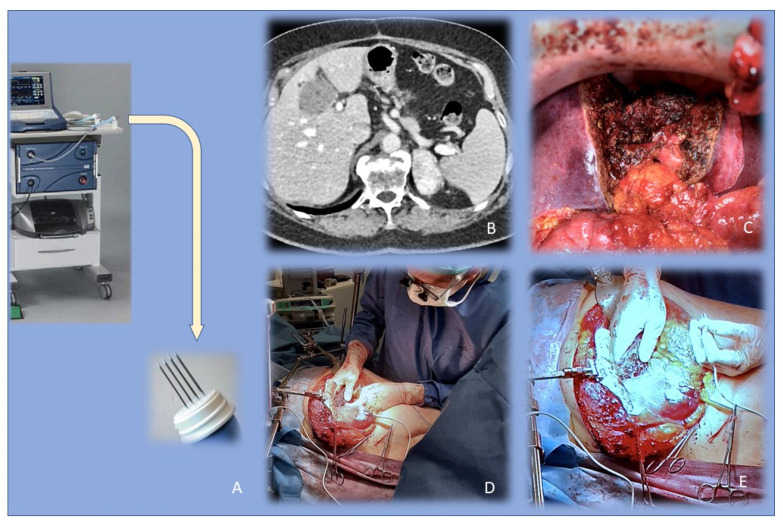
Electrochemotherapy (ECT) of deep-seated tumors. (**A**) equipment for intraoperative electroporation with magnified image of electrodes; (**B**) a large hilar cholangiocarcinoma, whose surgical complete removal was unlikely; (**C**) surgery was performed to remove all the macroscopical tumor burden; (**D**,**E**) chemoelectroporation was performed upon the perihilar liver tissue to improve the chance of cure.

More recently, thanks to the progress in the technical development, ECT has growing appeal in the treatment of deep-seated tumors (Figure 1). In this case, the technique is applied with simultaneous systemic (intravenous) administration of an anticancer drug, especially non-permeant bleomycin and poorly permeant cisplatin [59].

ECT is effective in the regression or resolution of hepatocellular carcinoma (HCC), and subjects’ quality of life after this treatment can improve [54]. ECT represents an important tool with hepatic metastases as well [52,60]. A recent phase II study proposed ECT in the treatment of unresectable colorectal liver metastases or for lesions untreatable by standard thermal ablative methods due to being located in the vicinity of major hepatic vessels [61]. Another phase II study assessed the safety of intraoperative ECT in the management of primary HCC [62]. An interesting in vivo study carried out on a murine model considered the use of ECT to treat prostate cancer. Bleomycin was injected into the tumor site 15 min before 10 pulses of 500 V/cm were delivered and its combination with ECT suppressed tumor growth [63]. In a preliminary study carried out on a series of patients with perihilar cholangiocarcinoma, ECT was shown to be a promising therapy to improve the prognosis and quality of life of patients affected by this kind of tumor [64]. A phase II clinical study was carried out on 29 patients affected by painful bone metastases. The results showed that ECT is safe and feasible in the treatment of painful bone metastases [65]. Cunha et al. reported the effects of ECT treatment on Ehrlich solid tumors in swiss mice. The animals tolerated ECT well, and the results achieved were encouraging [66]. ECT can be used as a palliative therapy in patients with loco-regional recurrence of squamous vulvar cancer (V-SCC). Perrone et al. treated nine patients (median age of 84 years) with V-SCC recurrence [67]. ECT was carried out after mapping of the lesions and bleomycin was intravenously injected under sedation. The results showed that ECT is a suitable procedure in elderly patients with V-SCC relapse, as ECT relieved symptoms and improved the quality of life. Analogous results were obtained with ECT palliative treatment of recurrent or metastatic vaginal cancers [68].

**Figure 2:**
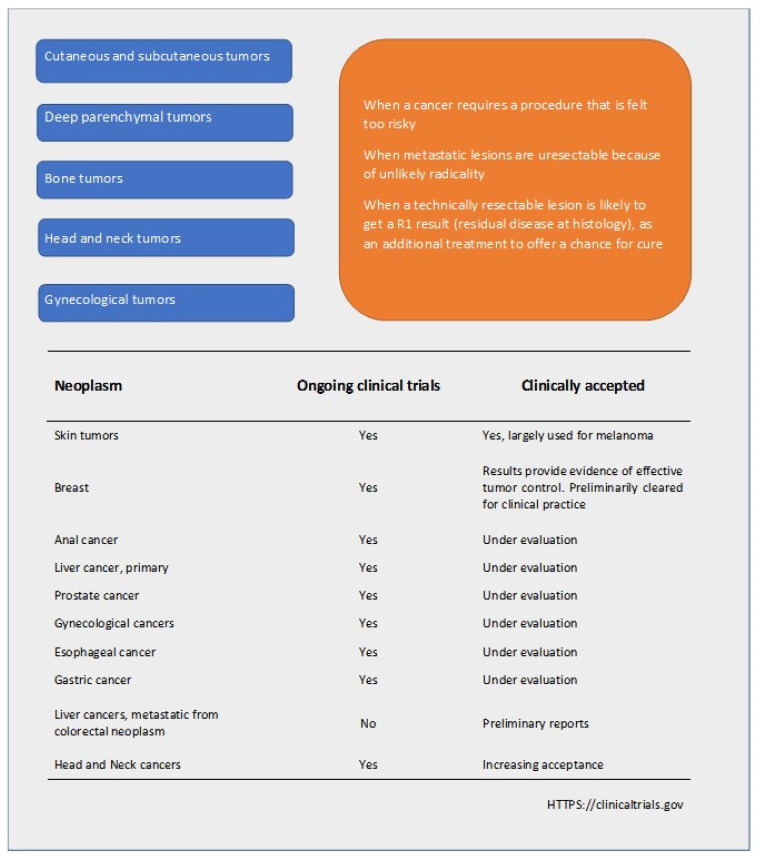
Scheme of the perspective clinical diffusion of electrochemotherapy (ECT) in antitumoral treatments.

Besides liver, pancreatic, and prostate cancers, ECT has shown feasibility and good efficacy in head and neck tumors, bone metastases, and gastrointestinal tumors [69]. Moreover, pioneering studies have identified lung and brain tumors as suitable future targets [68]. Furthermore, skin metastases, which can occur in 5–30% of breast cancer patients, can be treated with ECT using intravenous/intratumoral bleomycin or intratumoral cisplatin [70]. Figure 2 shows a summary scheme of ECT in the treatment of tumors.

Briefly, the requirements needed are an electroporator device and various electrodes, depending on the sizes of single or multiple tumoral nodules. In both IRE and ECT, the applied electric field is influenced by the electrical properties of the target tissues, the geometry and the position of electrodes, and the parameters of the electric pulses: amplitude, length, frequency, quantity, shape, and slopes [71]. ECT has been classified in long-needle variable electrode geometry ECT for the treatment, for example, of liver and pancreatic cancers, and endoscopic ECT, in the case of gastrointestinal tumors [68].

Finally, EP techniques can be considered safe and easy to perform [49]. They need systemic anesthesia, and in EP and ECT, irreversible breakdown of the membranes of the cells is undesirable.

## 3. Drugs Used in Electroporation-Based Therapies

As shown by the literature, different anticancer drugs have been studied for possible applications in ECT: carboplatin, cisplatin, doxorubicin, daunorubicin, actinomycin D, paclitaxel, adriamycin, mitomycin C, vinblastine, vincristine, 5-fluorouracil, gemcitabine, cyclophosphamide, sorafenib, and bleomycin. However, only two of these drugs are suitable candidates for ECT: bleomycin and cisplatin [48,54].

### 3.1. Bleomycin

**Figure 3:**
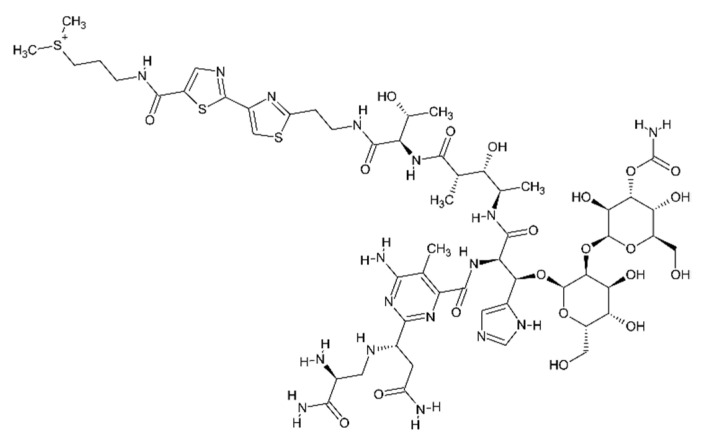
Bleomycin.

Bleomycin (Figure 3) is a water-soluble glycopeptidic antibiotic isolated from *Streptomyces verticillus* [72]. It is classified as a non-permeant drug [49]. Non-permeant drugs cannot pass across the cell membrane because of the characteristics of the molecule, such as the dimensions and/or physico-chemical properties.

A remarkable intrinsic cytotoxicity characterizes bleomycin because when the drug molecules come inside tumoral cells, they determine DNA single- and double-strand breaks, with DNA fragmentation, chromosomal gaps, and consequent mitotic death of the cells [49,73]. This effect is mediated by an oxygen- and metal ion-dependent process [73,74]. It has been calculated that only a few hundred molecules of bleomycin in the cytoplasm are sufficient to kill a cell [75]. However, this cytotoxicity is strongly inhibited by the failure of drug molecules to diffuse through the cell membrane and enter the cytoplasm [49,73]. It has been proved in vitro that without the EP effect, less than 0.1% of bleomycin, added to the extracellular medium, enters the cells [76].

Bleomycin transport through intact/non-permeabilized cell membranes occurs via endocytosis by carrier proteins; however, this process of transport is reduced by the low number of carrier proteins present on the cell surface [48,49,73]. EP increases membrane permeability and enhances the intracellular concentration of bleomycin, with an enhancement of the cytotoxicity against the cells of thousands of times [49]. The cytotoxicity regards the tumoral cells of the targeted tumor because this occurs when the cells try to divide, while quiescent cells stay alive [49]. Therefore, bleomycin is the drug most frequently combined with ECT.

Two different routes of administration of bleomycin are used: systemic (intravenous) or local (intratumoral). Thanks to the electropermeabilization effect, the dose of bleomycin necessary to obtain antitumor action is low enough to warrant a safe intravenous injection [49]. According to Mir’s opinion, who wrote an interesting review about the rational principles of ECT, the choice of systemic injection should be deemed an advantage because it allows, at the same time, the therapeutic treatment of multiple nodules [49], which, of course, must be located within the area subjected to the electric pulses. An intratumoral injection needs a minor quantity of drug; however, in the case of a multi-focal tumor, each nodule must be injected separately and consequently, the procedure is longer and less safe [49].

### 3.2. Cisplatin

**Figure 4:**
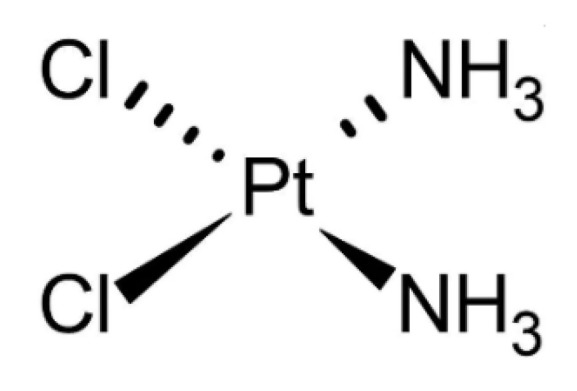
Cisplatin.

Cisplatin (Figure 4) is a platinum coordination complex that is used as an antineoplastic agent to treat various cancers. This drug is classified as low (poor) permeant [49,77]. This means it is a bit more permeable than bleomycin (which is non-permeant). In fact, about 50% of this drug crosses the cell membranes by passive diffusion, whereas the rest needs carrier molecules. Therefore, the overall flux through the membranes of the cells is merely limited by the presence of the carrier.

Cisplatin’s anticancer activity is based on two different mechanisms of action: cisplatin cross-links DNA and then inhibits DNA replication inducing cell apoptosis; and cisplatin adducts block the elongation of RNA and gene transcription, determining tumoral cell death [77,78].

It is reported that the increase in cisplatin’s efficacy due to ECT is lower than bleomycin [79]. Usually, this drug is used in the treatment of cutaneous metastases in humans, with intratumoral administration [80,81].

It is also reported that bleomycin is the drug most frequently used in ECT; however, in older patients or in patients affected by renal diseases, cisplatin should be preferred over bleomycin [82].

## 4. EP as a Potential “EPR Enhancer” for the Administration of Nanomedicines

The EP effects that occur when electric pulses are applied in a target tumor are complex and are not restricted solely to the permeabilization of the cell membranes.

At the end of the 1990s, pioneering works by Sersa et al. showed that besides the increased drug permeability through the cell membranes, the application of electric pulses influences the blood flow of tumors [83,84]. The effect of electric pulses on tumor blood flow was studied using the murine fibrosarcoma SA-1 model [83]. Tumor perfusion was determined using the ^86^RbCl extraction technique. After application of eight short intense electric pulses (1040 V) to the tumor, a significant reduction of tumor perfusion was found within 1 h, and the blood flow of the tumor slowly returned to the pretreatment level at about 24 h. It is also reported that the number of electric pulses applied influences the level of contraction of blood flow, as an inferior effect was found with less than eight pulses. In a later work, ECT of SA-1 subcutaneous tumors was carried out by the concomitant administration of cisplatin [84]. Here, the effect of ECT on the blood flow of the tumor was studied by the measurement of tumor perfusion with the Patent blue staining technique, and by determination of the tumor blood volume and microvascular permeability, using contrast-enhanced magnetic resonance imaging. After treatment by ECT with cisplatin, the tumor blood flow was reduced instantaneously, and this phenomenon was maintained until 24 h. This reduction determined a consequent reduced tumor oxygenation, and it was found that the cisplatin ECT treatment induced a delayed tumor growth of about nine days. The conclusions of the authors were that ECT’s efficacy is not simply due to increased cytotoxicity of cisplatin because of electropermeabilization of tumoral cells, but also due to reduced tumor blood flow and subsequent hypoxia.

Gehl et al. studied the vascular reactions following in vivo EP application in an animal model (C57Bl/6 mice) [85]. Pulses of 10–20,000 ms and 0.1–1.6 kV/cm were given on the hind- and forelimb of the animal and then a dye was injected to study the perfusion. Perfusion delays of about 1–2 min were recorded above the threshold and the authors claimed that a reflexory constriction of afferent arterioles caused these delays. This constriction was cut by over 50% when the mice were treated with reserpine, which is a blocking agent of the sympathetic nervous system, and is known to weaken regional vasoconstriction mediated by sympathetic fibers. Therefore, Gehl described the consequences of this phenomenon after EP as reflexory vasoconstriction of afferent arterioles mediated by the sympathetic nervous system and clearly defined the concept of “vascular lock”. In this paper, it was also marked out that these vascular effects that occur after EP application could “affect kinetics of drug delivery”. In fact, this “vascular lock” reduces drug washout and consequently clearance, and this effect could be relevant in the case of direct intratumoral drug injection.

The effects of EP applications on blood vessels were also studied by Bellard et al. in an interesting work that was carried out in C57Bl/6 mice [86]. The study was carried out by direct visualization, using intravital microscopy in a mouse dorsal window chamber (DWC) model. This work suggested the complexity of the effects that take place after the application of electric pulses to a target tumor. The authors noticed that the application on the animal skin of electric pulses determined both a swift improvement of vascular permeability (“extravasation effect”) that gradually bounced back to the basal level, and simultaneously, a prompt constriction of the blood vessels, which was more noticeable in arterioles with respect to venules, and which determined a reversible “vascular lock” that lasted a maximum of 10 min. Furthermore, the authors studied the behavior of fluorescently labeled dextrans of different sizes administered systemically by intravenous injection. They found that, whatever the dextran size, there was an increased permeability of the molecules through the small vessel walls, associated with the delayed perfusion.

A similar in vivo study was carried out using HT-29 human colorectal adenocarcinoma cells implanted in SCID mice. The response of blood vessels to electric stimulation was recorded by in vivo optical imaging (intravital microscopy) in a dorsal window chamber, with fluorescently labeled dextrans (70 kDa) [87]. The electroporation techniques used were both EP and ECT, using bleomycin as a model drug in the latter case. Three minutes before EP, bleomycin solution (in sterile aqueous solution) was injected in the retro-orbital plexus. The authors noted that both EP and ECT determined the “vascular lock”. They also found an increased blood vessel size and decreased so-called “functional vascular density” (FVD) in the tumor’s body. They found that, in the conditions in which the experiments were carried out, bleomycin-based ECT treatment destroyed the blood vessels of the tumor within 24 h, adding to the state of hypoxia. The results of this work corroborate those regarding the reduced tumor blood flow and consequent hypoxia found by Sersa et al. using cisplatin [84]. The observed vessel damage attributed to the administration of bleomycin could seem to contradict the results obtained by other researchers, as previously reported [52,56], which did not find any damage connected with drug administration. However, the contradiction could only be apparent because there is clearly a dependance of this phenomenon on the drug dose.

Even if these studies refer to macromolecules and not nanoparticles, these results encourage study of the behavior of nanocarriers present in a target tumor during and after the application of electric pulses.

Srimathveeravalli et al. administered ^89^Zr-radiolabeled liposomes via the intravenous route to tumor-bearing mice (implantation of MiaPaca-2 cells) [88]. The ^89^Zr-radiolabeled liposomes were used to monitor ECT administration of doxorubicin-loaded liposomes (pegylated liposomal doxorubicin, Doxil). EP application resulted in improved uptake of the ^89^Zr-radiolabeled liposomes. The authors observed that the radiolabeled liposome injection before the EP application determined an immediate increase in liposome uptake, while this rapid increase was not observed when the injection was performed after the application. However, the sequence of ^89^Zr- radiolabeled liposome injection and electric pulse application did not determine a significant difference in the total final tumor uptake. In fact, at 24 h, no differences were found in the uptake between tumors treated with injection before EP or injection after EP. According to the authors’ opinion, this means that EP’s effects on tumoral blood vessels have the most important role in the uptake of nanocarriers into tumors, long after the reversible effect of membrane permeability has disappeared. They explicitly affirmed that when EP is applied to the tumor mass, modifications of the endothelium, combined with the “vascular lock” and the permeabilization effect of cell membranes, modify the EPR effect of the tumor. These authors stated the importance of a full understanding of the complex effects of EP. Their conclusion was that the correlation between EP’s effects and the EPR effect of the tumors needs further studies.

Kodama et al. wrote an interesting paper about EP-induced changes in the tumor vasculature and microenvironment and the improvement of the efficacy of sorafenib nanoparticles [89]. Dye-stabilized sorafenib nanoparticles were obtained by using a nano-precipitation method. Human endothelial (EA.hy926) and colorectal carcinoma (HCT116) cell lines were used. EA.hy926 endothelial cells were grown in monolayers. The endothelial monolayer morphology and permeability were studied following EP application. HCT116 cells were seeded in ultra-low attachment cell culture flasks to allow their aggregation in tumorspheres that were about 200–300 μm in size. The penetration of nanoparticles into tumorspheres, after EP application, was imaged with fluorescent microscopy. Female athymic NU/NU nude mice were implanted with colorectal carcinoma (HCT116) cells to obtain bilateral flank tumors that grew to 5–7 mm in size. Mice (*n* = 5) with bilateral flank tumors underwent treatment of one tumor with EP (1000 V/cm, 100 μs, 1 Hz, 8 pulses) while the contralateral side served as the control. The experimental results showed the EP increases the transport of nanoparticles through endothelial monolayers, promotes the penetration and accumulation of nanoparticles into tumorspheres, and ultimately strengthens the uptake of sorafenib nanoparticles in cancer cells. The in vivo experiments carried out in the mice with bilateral tumors showed that EP promotes nanoparticle delivery to the tumors and can improve the therapeutic efficacy of sorafenib nanoparticles. The authors claimed that EP has simultaneous effects on the tumor cell membrane, microvasculature, and extracellular space. Thus, it is a tempting candidate technique as a promoter of nanoparticle-based cancer therapy. The preliminary results of this work are impressive because they reinforce the rationale about the possible use of the EP technique as an “EPR enhancer” of nanoparticles.

Kulbacka et al. studied the value of the EP technique combined with solid lipid nanoparticles (SLNs) incorporating a cyanine-type diagnostic/photosensitizer agent, IR-780, and a flavonoid derivative, baicalein or fisetin [90]. IR-780 is a cyanine derivative, which can be considered as second generation, used in photodynamic therapy (PDT). The study was carried out using two cell line models: human colon adenocarcinoma (LoVo) and hamster ovarian fibroblastoid (CHO-K1). The intracellular accumulation of cyanine IR-780 was visualized with the confocal microscopy method. EP was carried out with free or encapsulated IR-780. The experimental results showed increased IR-780 uptake, much higher in the case of SLNs with respect to free cyanine. According to the authors’ opinion, the combination of EP with SLNs can be successfully applied for improved photodynamic (PDT) therapy, thanks to the enhanced SLN uptake into the tumor.

Lin et al. tested the addition of curcumin loaded into hydrophobically modified glycol-chitosan nanoparticles combined with EP application (8, 1200 V/cm, 100 μs electrical pulses) on human breast carcinoma cells (MCF-7) and compared it with free curcumin treatment [91]. The nanoparticles consisted of glycol-chitosan conjugated with β-cholanic acid. The results showed that the EP condition used did not result in irreversible damage of the cells. EP seemed to enhance the cell numbers in the presence of serum, and this was probably because of the increased uptake of nutrients in the serum. Under the EP application, curcumin-loaded nanoparticles were much more effective in suppressing the growth of tumor cells compared to free curcumin. Curcumin-loaded nanoparticles were also more effective in inducing apoptosis, compared to free curcumin.

Phonesouk et al. published a paper in which they describe an increased uptake of fluorescently labelled silica nanoparticles (SiNPs, 28–30 nm) with EP [92]. SiNPs represent an important nanomaterial that is biocompatible [93] and biofunctionalizable [94], with great potential for applications in the field of drug targeting/delivery and theranostics. The uptake of SiNPs by cells usually occurs via endocytosis, which, however, is difficult in certain cases, and SiNPs are only distributed in the cytoplasm and never reach the nucleus. This preliminary study was carried out using different cancer lines: HCT116 (human colon cancer) cells and RL (follicular lymphoma) cells. The results showed that upon application of EP, improved and more efficient penetration of SiNPs into the cells occurred, reaching the nucleus. The entry of SiNPs into the cells was fast. These preliminary results open perspectives of an improved use of SiNPs combined with EP to prepare smart nanocarriers for the diagnosis or treatment of tumors. According to the authors’ opinion, the penetration of the nanoparticles into the nucleus of the cell could be a significant aspect of this uptake being “EP-improved” because nucleus-targeted drug delivery opens new and crucial strategies for anticancer therapy.

## 5. Conclusions

The discovery of the EPR effect represented the hope for the development of specific nanocarriers, administered systemically, for therapy and/or diagnosis of targeted tumors. However, in humans, the EPR effect is variable, heterogeneous, and “below expectations”, with consequent unpredictable uptake of the nanocarriers by the tumor. The discussion of the EPR effect in the field of antitumoral nanomedicine is open.

Therefore, in the field of anticancer therapies, there is a growing consensus about the strong need for “EPR enhancers” to improve the performances of nanocarriers.

Electroporation techniques are well-known multifunction tools for anticancer treatments. They are used as nonthermal ablation techniques, or for the delivery of non-permeant/low-permeant drugs into the targeted tumors. The technique is usually combined with intravenous (systemic) or intratumoral (local) administration of (free) drug solutions. The effects determined by the application of the electric pulses in the target tumoral tissue are complex and involve, besides cell membrane permeabilization, a reversible vascular block and an improvement of the permeability of blood vessels.

**Figure 5:**
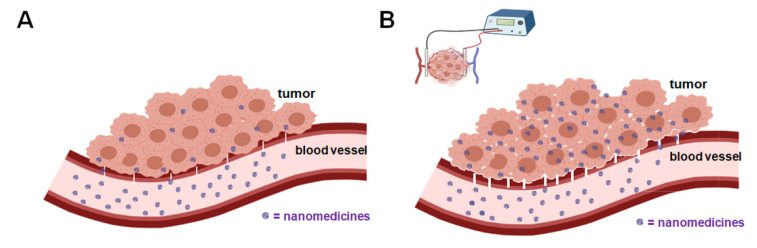
(**A**) Nanomedicine and EPR effect; (**B**) nanomedicine and EPR-enhanced effect by application of electroporation.

The problem of finding new strategies to achieve EPR enhancement is the topic of many papers. The complex modifications that occur in the target tumor during the application of electric pulses could improve the EPR effect, if combined with concomitant intratumoral administration of nanocarriers (Figure 5).

Electroporation combined with the local administration of nanomedicines is worthy of further investigations due to its influence on the EPR effect. The possibility of modulating local clearance, influenced by reduced blood flow, combined with administration of drug-loaded nanocarriers may improve their targeting into permeabilized tumor cells.

Liver and pancreatic tumors can be considered as good candidates for anticancer therapies based on the combination of electroporation and intratumoral injection of nanocarriers because this technique can be applied as a complement to surgery or alternatively, in the case of inoperable tumors.

**Figure 6:**
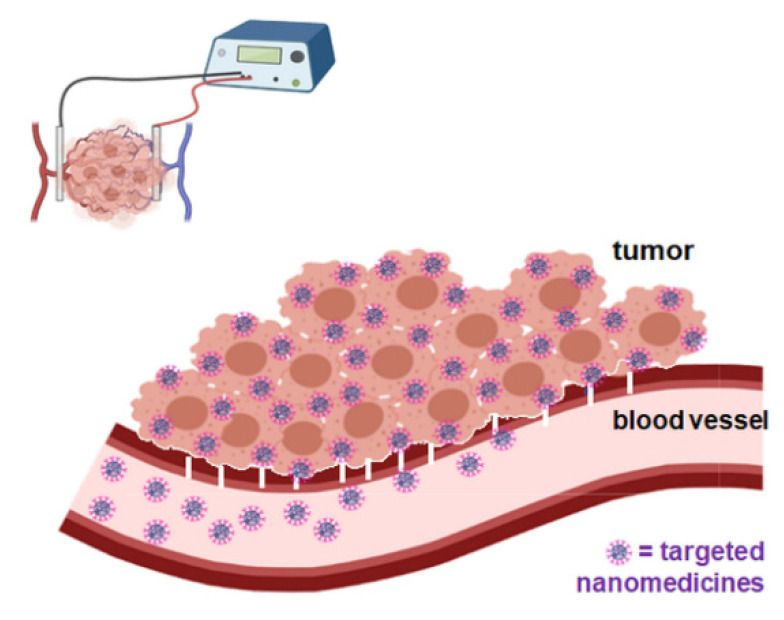
Targeted (functional ligand) nanomedicine and the EPR-enhanced effect by the application of electroporation.

Furthermore, active targeting based on surface receptors on target cells has been widely explored since malignant cells upregulate specific surface structures that can be targeted by nanoparticles as recognition sites. Local application of active targeting, represented by the intratumoral administration of nanocarriers conjugated with functional ligands for tumor receptors, could be used as a complementary strategy to EPR-based passive targeting, to improve nanomedicine accumulation and the retention of multinodular tumors, with one single injection (Figure 6). Additionally, this approach could take advantage of the enhanced EPR effect by EP application.

Therefore, the use of electroporation techniques to improve nanomedicine uptake by tumors appears to be promising and can have wide potential applications in cancer therapy in the future.

## Figures and Tables

**Table 1:** EPR enhancers to obtain an “EPR-adaptive delivery” in nanomedicine.

Kind of Technique	Enhancer	Reference
Pharmacological co-treatment	Angiotensin II-induced hypertension	[31]
Pharmacological co-treatment	NO-generating agents	[32]
Pharmacological co-treatment	CO generating agents	[33]
Physical co-treatment	Ultrasound	[30]
Physical co-treatment	Radiation	[30]
Physical co-treatment	Hyperthermia	[30]
Physical co-treatment	Photodynamic therapy	[30]
